# Gangliosides: Treatment Avenues in Neurodegenerative Disease

**DOI:** 10.3389/fneur.2019.00859

**Published:** 2019-08-06

**Authors:** Pierre J. Magistretti, Fred H. Geisler, Jay S. Schneider, P. Andy Li, Hubert Fiumelli, Simonetta Sipione

**Affiliations:** ^1^Biological and Environmental Science and Engineering Division, King Abdullah University of Science and Technology, Thuwal, Saudi Arabia; ^2^Brain Mind Institute, École Polytechnique Fédérale de Lausanne (EPFL), Lausanne, Switzerland; ^3^Department of Psychiatry, Center for Psychiatric Neurosciences, Lausanne University Hospital (CHUV), Lausanne, Switzerland; ^4^Department of Medical Imaging, College of Medicine, University of Saskatchewan, Saskatoon, SK, Canada; ^5^Parkinson's Disease Research Unit, Department of Pathology, Anatomy and Cell Biology, Thomas Jefferson University, Philadelphia, PA, United States; ^6^Department of Pharmaceutical Sciences, Biomanufacturing Research Institute Technology Enterprise (BRITE), North Carolina Central University, Durham, NC, United States; ^7^Department of Pharmacology, Neuroscience and Mental Health Institute, University of Alberta, Edmonton, AB, Canada

**Keywords:** Parkinson, Huntington, Alzheimer, spinal cord injury, stroke, GM1, neuroprotection, glia

## Abstract

Gangliosides are cell membrane components, most abundantly in the central nervous system (CNS) where they exert among others neuro-protective and -restorative functions. Clinical development of ganglioside replacement therapy for several neurodegenerative diseases was impeded by the BSE crisis in Europe during the 1990s. Nowadays, gangliosides are produced bovine-free and new pre-clinical and clinical data justify a reevaluation of their therapeutic potential in neurodegenerative diseases. Clinical experience is greatest with monosialo-tetrahexosyl-ganglioside (GM1) in the treatment of stroke. Fourteen randomized controlled trials (RCTs) in overall >2,000 patients revealed no difference in survival, but consistently superior neurological outcomes vs. placebo. GM1 was shown to attenuate ischemic neuronal injuries in diabetes patients by suppression of ERK1/2 phosphorylation and reduction of stress to the endoplasmic reticulum. There is level-I evidence from 5 RCTs of a significantly faster recovery with GM1 vs. placebo in patients with acute and chronic spinal cord injury (SCI), disturbance of consciousness after subarachnoid hemorrhage, or craniocerebral injuries due to closed head trauma. In Parkinson's disease (PD), two RCTs provided evidence of GM1 to be superior to placebo in improving motor symptoms and long-term to result in a slower than expected symptom progression, suggesting disease-modifying potential. In Alzheimer's disease (AD), the role of gangliosides has been controversial, with some studies suggesting a “seeding” role for GM1 in amyloid β polymerization into toxic forms, and others more recently suggesting a rather protective role *in vivo*. In Huntington's disease (HD), no clinical trials have been conducted yet. However, low GM1 levels observed in HD cells were shown to increase cell susceptibility to apoptosis. Accordingly, treatment with GM1 increased survival of HD cells *in vitro* and consistently ameliorated pathological phenotypes in several murine HD models, with effects seen at molecular, cellular, and behavioral level. Given that in none of the clinical trials using GM1 any clinically relevant safety issues have occurred to date, current data supports expanding GM1 clinical research, particularly to conditions with high, unmet medical need.

Gangliosides are a cell membrane component ubiquitous in vertebrates and most abundant in the central nervous system (CNS). Gangliosides are glycosphingolipids composed of a ceramide base with an oligosaccharide chain to which one or more sialic acids are bound. Description of the structure and the biosynthesis steps of the major gangliosides can be found in recent reviews (1, 2). Among over 60 known natural gangliosides, monosialo-tetrahexosyl-ganglioside (GM) 1, disialo-gangliosides GD1a and GD1b, and trisialo-ganglioside GT1b are the most common ones, with GM1 accounting for ~28% of the total human brain gangliosides (3). Although gangliosides are known for about 75 years, much of their role is still unknown and the research interest in their diverse functions remains high, as demonstrated by about 400–500 articles published in scientific journals worldwide every year.

Gangliosides have extensively been tested in diverse clinical applications. Until the early 1990s, a ganglioside extract produced from calf brains was marketed in Europe as treatment for acute or chronic CNS lesions and Parkinson's disease (PD) (e.g., Cronassial® in Germany, Nevrotal® in Spain, and Sygen® in Italy). Eventually, the product was withdrawn from the European market after reports of Guillain-Barré-Syndrome (GBS), a rare misdirected immune response to gangliosides causing peripheral nerve damage, often following infections. Noteworthy, this withdrawal coincided in Europe with the peak time of bovine spongiform encephalopathy (BSE) through a newly discovered type of infection caused by prions, raising scrutiny for any human use of products derived from bovine brain. Meanwhile, bovine-free ganglioside products were developed from porcine brain material. BSE has never been documented in pigs and is almost eradicated in 2019, limiting the potential for a spread across species. Neither epidemiological studies (4, 5) nor post-marketing safety data in over 1 million patients exposed to a GM1-product from porcine brain worldwide support the incidence of GBS to be associated with GM1 use. Additionally, the injection of GM1 alone had no immune-stimulant effects (6) and no anti-GM1 antibodies were detected after long-term treatment with GM1 doses of 1,000 mg i.v. followed by 200 mg/day s.c. for 18 weeks (7).

Recently, gangliosides have been proposed to play a key role also in cancer (8), diabetes (9), and infection (10). However, while these indications currently have effective treatments, therapy of many degenerative neurological diseases has not progressed much. The medical need for new treatments of neurodegenerative conditions continues to increase in aging populations and, particularly in those with orphan status, remains largely unmet. In parallel, preclinical evidence of potentially beneficial GM1 effects in such indications has evolved. Therefore, further clinical testing of GM1 in some neurological indications may warrant a reevaluation.

## Mechanisms of Action

Gangliosides play an important role in the development, protection, and repair of the CNS (1, 11, 12). Not surprisingly, genetic defects that affect their synthesis result in severe early-onset neurological diseases (13). Mutations in the *ST3GAL5* gene, which encodes the first sialyltransferase (GM3 synthase) in the ganglioside biosynthetic pathway, cause an early-onset epilepsy syndrome with severely delayed motor and cognitive development and choreoathetosis. Blindness and deafness are also present in most patients (14). Mutations in *B4GALNT1*, which codes for GM2/GD2 synthase, are linked to a form of hereditary spastic paraplegia characterized by limb spasticity, dysarthria, peripheral neuropathy, and severe intellectual disability (15, 16).

Besides these rare diseases, changes in the ganglioside profile (i.e., in the relative abundance of specific gangliosides) were reported in degenerative CNS conditions, including Alzheimer's (AD) (17, 18), PD (19), Huntington's disease (HD) (20, 21), multiple sclerosis (MS) (22, 23), and amyotrophic lateral sclerosis (ALS) (24). GM1 deficiencies in particular have been detected in PD (19) and HD (20), whereas GM1 expression and distribution were shown to be affected in CNS injury caused by trauma or disease (25, 26). GM1 is one of the predominant brain gangliosides (3), with demonstrated anti-neurotoxic, neuroprotective, and neurotrophic actions *in vitro* and *in vivo* (27–29).

Early studies suggested that the action of gangliosides is closely related to that of neurotrophins, as they display similar neuroprotective effects and modulate neurotrophin signaling (29, 30). This is supported by the ability of GM1 to facilitate the activation of tropomyosin-related kinase (Trk) receptors and the signaling cascade downstream, as well as the induction of neurotrophin synthesis and release (31–33). The neurotrophin family in mammals comprises 5 members, i.e., the nerve growth factor, the brain-derived neurotrophic factor (BDNF), and the neurotrophins 3, 4, and 5 (34). All neurotrophins promote survival of subpopulations of neurons in the central and the peripheral nervous system, but with different specificity, i.e., the potency of protective effects deviates for different subpopulations of neurons.

**Figure 1:**
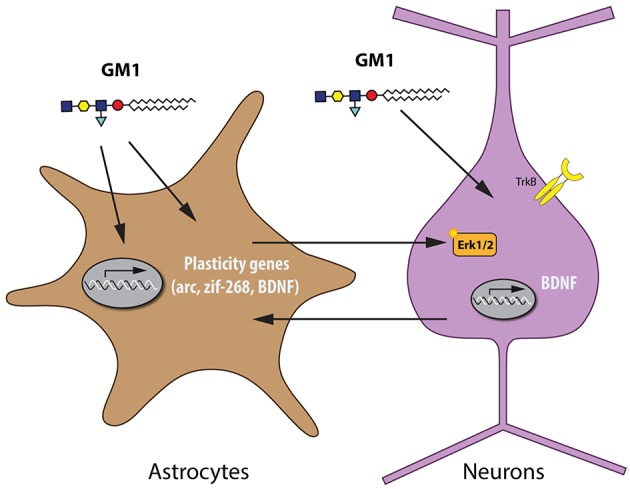
Astrocytes are required for the activation of the MAPK pathway by GM1 in neurons.

The neuroprotective profile of GM1, as shown in experimental models of spinal cord injury (SCI), PD, stroke, HD, and AD, is reminiscent of the actions of BDNF. BDNF has key neurotrophic and neuroprotective functions in the developing and adult brain, which makes it a potential tool for many therapeutic strategies (35), e.g., BDNF was shown to protect against tau-related neurodegeneration in a mouse model of AD (36). GM1 stimulates release of BDNF (37) and acts synergistically with BDNF (38). When BDNF binds to its receptor TrkB, it triggers the mitogen-activated protein kinase (MAPK) pathway, which mediates neurotrophic effects such as dendritic growth (39, 40). In recent studies in cultured rat cortical neurons, GM1 did neither stimulate BDNF synthesis or release, nor BDNF/TrkB signaling pathways. In mature and more complex brain preparations such as cortical prisms from adult mice, however, GM1 stimulated the MAPK pathway. In mixed cultures and co-cultures of various ages, GM1 activated the MAPK pathway in mature cultures, but only when astrocytes were present (41). These findings indicate that GM1 can activate similar pathways as BDNF, which has key neuroplastic and neuroprotective roles in the adult brain. Recent evidence has been provided that astrocytes can also be a source for BDNF and that possibly a bidirectional transfer of BDNF between astrocytes and neurons can be considered (42, 43), indicating that the presence of astrocytes is required for this effect (Figure 1).

Additional mechanisms are likely to contribute to the protective effects of GM1 across different diseases. These could include a modulatory role of the ganglioside on ion channels and/or neuronal Ca^2+^ homeostasis (44–47). These and other potential neuroprotective roles of gangliosides have been reviewed elsewhere (29, 48) and will not be discussed further here. GM1 may have broader therapeutic potential than thought in the 1990's. To further elucidate the action of GM1 in the brain, its effect on gene expression in primary cultures of rat astrocytes and neurons was studied. Transcriptome analysis with next-generation sequencing (49) was used to determine the differential expression of genes under different conditions. Significant results were selected based on a ≥±1.5-fold change in expression. A large number of differentially regulated genes with GM1 vs. control were found: 291 in pure cultures of astrocytes, 800 in astrocytes co-cultured with neurons, 78 in pure cultures of neurons, and 1,719 in neurons co-cultured with astrocytes. Further studies are ongoing to identify major pathways regulated by GM1 in neurons and astrocytes.

## Therapeutic Potential in Neurological Indications

Although many of the molecular mechanisms through which GM1 may exert neuroprotective actions remain unknown, those actions are quite evident, *in vitro* and *in vivo*, in animals as well as in humans, as demonstrated in the following key neurological indications.

### Spinal Cord Injury

SCI is an indication with orphan designation in Europe and the US. The incidence of SCI in the US is approximately 54 cases per million or approximately 17,000 new cases each year (50). Although estimations of the annual incidence and prevalence vary by country and region (51, 52), the World Health Organization estimates the annual global incidence at 40-80 cases per million, with 250,000–500,000 people worldwide suffering from SCI every year (53). Most SCIs are traumatic in origin; affected patients are often young and remain severely disabled for the rest of their lives. Thus, the economic and social burden caused by SCI is enormous (54).

Current medical management of SCI is mechanical decompression and restoration of normal blood pressure to correct the low perfusion to the injured spinal cord tissue within 4 h post-injury and by this to limit secondary injury. Ideally, at about 24 h post injury medical management would add neurotrophic and regenerative therapy to block neuronal death. Currently, several neuroprotective and regenerative agents are in clinical development (55, 56), but none has obtained US FDA clearance or scientific community acceptance yet. Although the US FDA never approved high-dose methylprednisolone (MPSS) for the treatment of SCI, it has broadly been used as a neuroprotective treatment since the 1990s, based on the US NIH recommendations following the second National Acute Spinal Cord Injury Study (NASCIS II) (57). This study was a large NIH funded, multicenter, double-blind, randomized, controlled trial comparing the efficacy and safety of MPSS and naloxone vs. placebo. Following the subsequent NASCIS III study, the US NIH added further recommendations for patients to be maintained on MPSS for either 24 or 48 h, depending on whether treatment was initiated within 3 or within 3–8 h post-injury, respectively (58).

Prior to the initial scientific publication of the NASCIS II in the NEJM, abbreviated lay study conclusions were disseminated via media, press release, and even the unprecedented step of sending a US NIH clinical alert to all US emergency rooms to start MPSS therapy in SCI patients. This massive dissemination of the claimed results of the NASCIS II study quickly established the use of MPSS in common practice in the US. However, it soon became apparent that the NASCIS II design was flawed and that the statistical analysis was incomplete and poorly reported. At least 11 peer reviewed articles on such issues were published (59) and acknowledged by the American College of Surgeons (60). In two Cochrane reviews, the latest in 2012, the main authors of the NASCIS studies still concluded though, that high-dose MPSS therapy was “*the only pharmacologic therapy shown to have efficacy in a phase-III randomized trial when administered within 8 h of injury*” and “*additional benefit by extending the maintenance dose from 24 to 48 h, if start of treatment must be delayed to between 3 and 8 h after injury*” (61, 62). However, in addition to the criticism of methods used in the NASCIS trials, CRASH (Corticosteroid Randomization After Significant Head injury), a randomized, placebo-controlled trial in 10,000 adults with head injury and Glasgow Coma Scale score of ≤14, revealed a higher risk of death in patients treated with the recommended MPSS regimen as compared to those treated with placebo (relative risk: 1.15, 95% CI 1.07–1.24; *p* = 0.0001). The CRASH results strongly discouraged further routine use of MPSS in any trauma patients (63) including those with SCI. It is noteworthy that the NASCIS studies required an initial neurologic examination from a cooperative patient, so acute SCI trauma patients unconscious from a head injury or intubated from chest trauma were excluded. This explains why the NASCIS studies did not reveal any negative MPSS drug effect on acute SCI trauma patients.

As a consequence of the CRASH results, evidence-based treatment guidelines no longer recommended routine use of high-dose MPSS for the treatment of SCI in the US (64, 65). Based on the flaws in design, presentation, analysis, and interpretation of results, the NASCIS trials were classified by Neurosurgery Guidelines as providing level III evidence at best (65). By contrast, two trials conducted with GM1 in acute traumatic SCI at about the same time were considered to provide level I evidence (64–66). In a single-center, double-blind, randomized, placebo-controlled, pilot study, 37 patients with SCI were enrolled to evaluate the efficacy of daily intravenous doses of 100 mg GM1 given for 18–32 doses starting within 72 h post-injury in enhancing the functional recovery of damaged neurons over a 1-year follow-up period (67, 68). Neurologic recovery was assessed using the 5-point Frankel score and the American Spinal Injury Association (ASIA) motor score. Patients treated with GM1 showed superior improvement vs. placebo in both these scores over 1 year.

**Figure 2:**
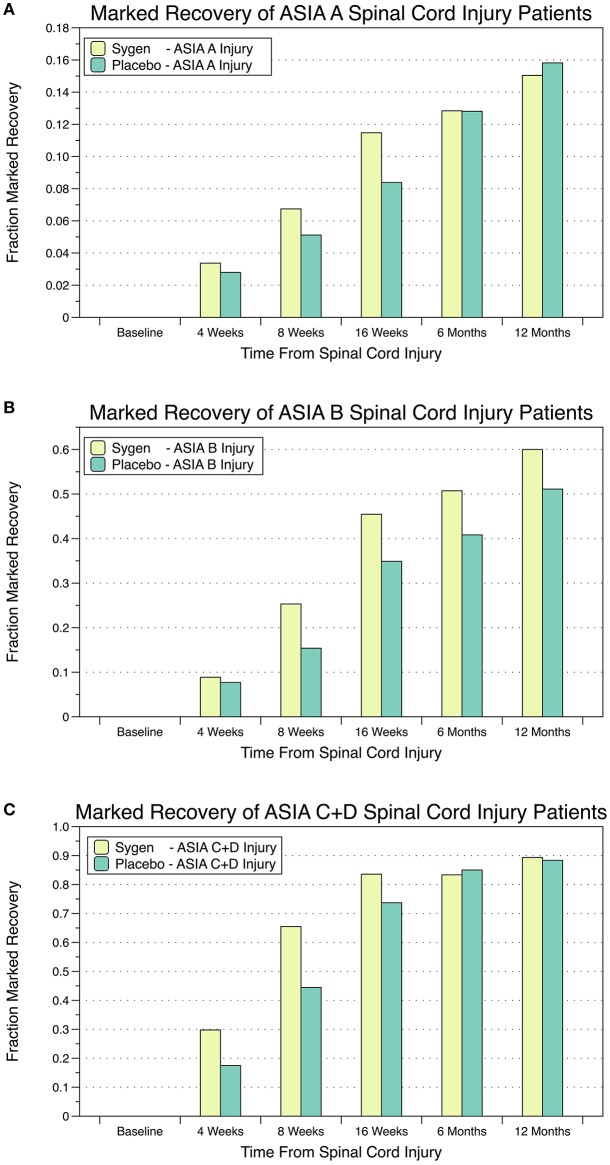
Percentage of SCI patients with marked recovery, i.e., ≥2-grade improvement from the entry ASIA Impairment Score to the modified 7-point Benzel classification during follow up. **(A)** Patients with complete SCI and no motor or sensory function preserved; **(B)** Patients with incomplete SCI and sensory but no motor function preserved; **(C)** Patients with incomplete SCI and motor function preserved below the neurological level.

Based on these encouraging results, a large multicenter, double-blind, randomized, placebo-controlled trial was launched testing two dose regimens of GM1 vs. placebo in 760 patients with acute SCI (69). Eligible patients had to have major SCI with a neurological deficit in one lower limb and a total ASIA motor score **≤**15, without cord transection or penetration, cauda damage, significant plexus, or peripheral nerve injury. After completion of MPSS treatment within 8 h post-injury, patients were block-randomized to receive either placebo or GM1, at high (600 mg initially, followed by 200 mg/day i.v.) or low doses (300 mg initially, followed by 100 mg/day i.v). Randomization was stratified by the level of injury (cervical vs. thoracic), baseline ASIA Impairment Score (complete: no motor or sensory function preserved [A]; incomplete with sensory but no motor function preserved [B]; incomplete with motor function preserved [C+D] below the neurological level), and age (<29 vs. >29 years). Treatment started within 72 h post-injury and lasted for 8 weeks. The main efficacy measure was the fraction of patients achieving at least a 2-grade improvement from the entry ASIA Impairment Score to the modified 7-point Benzel classification during follow up (Figure 2) (70). This classification by marked recovery allowed using one common binary assessment of neurologic function. In 28 centers, 3,130 patients were screened of whom 797 were eligible and randomized, and 760 were analyzed for efficacy. In 482 patients SCI was complete (ASIA Impairment Score: A), in 278 incomplete (B-D), in 579 the lesion was at cervical and in 181 at thoracic level; 395 patients had cervical traction and 600 patients a spinal operation. The study population was typical for SCI patients in the distribution of neurological level, age, and sex. Median times from injury to MPSS treatment was <2 h. There were no relevant differences among treatment groups at baseline. At 8 (end of treatment) and 16 weeks, the proportion of patients with marked recovery, i.e., ≥2 grade improvement in the modified Benzel classification was significantly greater in both GM1 dose groups as compared to placebo (Figure 2). This difference in neurologic recovery between treatment groups was no longer present at week 26, the primary endpoint. Thus, the study demonstrated a significantly faster, although ultimately not greater recovery with GM1 therapy. Detailed analysis demonstrated that the partial SCI patients had the most pronounced acceleration in recovery rate (Figure 2C). The ASIA motor, light touch, and pinprick scores as well as bowel and bladder function, sacral sensation, and anal contraction all showed a consistent trend in favor of GM1 enhancing neurologic recovery (69–71).

Also in the literature were three rather small, double-blind, randomized, placebo-controlled trials demonstrating faster recoveries through GM1 in patients with chronic SCI (72), disturbance of consciousness after subarachnoid hemorrhage (73), or craniocerebral injuries due to closed trauma (74). None of these trials reported any clinically relevant safety issue with GM1.

### Stroke

By far the greatest clinical experience with GM1 exists in the treatment of stroke with overall 14 double-blind, randomized, placebo-controlled, clinical trials in overall >2,000 patients (Table 1). The 4 largest trials enrolled 792, 502, 287, and 99 patients with acute ischemic stroke within 5, 12, 48, and 48 h from its onset, respectively (75–78). In 2 trials, higher i.v. loading doses of 300 and 200 mg were used (75, 78), otherwise GM1 was given in all studies at constant dose levels of either 40 or 100 mg and either i.v. or i.m. for 2–6 weeks. Patients were followed for 3–6 months.

**Table 1:** Double-blind Randomized Clinical Trials (RCTs) on the efficacy and safety of GM1 in patients with acute stroke in the order of decreasing size.

					**Efficacy measures**						
**References**	***N* of patients**	**GM1 dose [mg/day]**	**Treatment duration**	**Follow-up**	**Neurological assessment**	**Barthel scale**	**CT**	**EEG**	**PET**	**Survival**	**Other scales**
Lenzi et al. (75)	792	300 i.v. (d1), 100 i.v. (d2–10), 100 i.m. (d11–21)	3 w	4 m						X	Canadian neurological
Argentino et al. (76)	502	100 i.v. ± hemodilution	2 w	4 m						X	Rankin, modified; Canadian neurological
Alter et al. (77)	287	100 i.m.	4 w	3 m		X				X	Toronto stroke
Scarpino et al. (78)	99	100/200 i.v. (d1), 100 i.v. (d2–21)	3 w	6 m		X					Fritz-Werner
Hoffbrand et al. (79)	49	100 i.m.	4 w	6 m	X	X					
Reuther et al. (80)	42	100 i.v.	3 w	3 m	X	X					
Battistin et al. (81)	40	40 i.m.	6 w	6 w	X		X	X			
Bassi et al. (82)	38	40 i.m.	6 w	6 w			X	X			Mathew
Frattola (83)	38	40 i.m.	6 w	6 w	X		X	X			
D'Agnini and Cesari (84)	37	40 i.m.	6 w	6 w	X		X	X			
Jamieson et al. (85)	30	40 i.v.	n.s.	6 m			X		X	X	
Heiss et al. (86)	25	100 i.v.	3 w	3 m	X	X	X		X		Stroke index
De Blasio et al. (87)	20	100 i.v.	10 d	10 d							Glasgow-pittsburg coma, acute stroke, modified
Abraham and Lange (88)	19	100 i.m.	4 w	3 m	X	X					

*CT, computed tomography; d, day; EEG, electroencephalogram; i.m., intramuscular; i.v., intravenous; m, month; PET, positron emission tomography; s.c., subcutaneous; w, week*.

In 3 of these largest 4 trials survival was analyzed, but none found any difference vs. placebo. Methods for the neurological evaluation varied among the four trials and only the Canadian Neurological and the Barthel scale were used more than once, i.e., each of them in two trials. In the largest study (75), the improvement from baseline in the Canadian Neurological score was greater with GM1 than with placebo, but only in the subgroup of patients treated within 4 h from stroke onset, the difference was significant. In the second study (76), among 427 patients who presented with a first ischemic hemispheric stroke, a significantly greater neurological improvement was found vs. placebo at the end of treatment. In the third study (77), the Toronto Stroke scale and Barthel Index showed no significant difference vs. placebo on Day 84. However, the difference in the improvement of the motor component of the Toronto Stroke scale was significant on Day 28 and still in favor of GM1 on day 84, as were all 10 components of the Barthel Index. In the last study (78), significant differences were found vs. placebo for both the Fritz-Werner and the Barthel Index on Day 21 which for the Barthel Index persisted for 6 months. Overall, all 4 studies demonstrated benefits through GM1 vs. placebo in neurological outcomes.

In 10 additional, smaller studies with <50 patients per trial, GM1 was given at either 40 or 100 mg/day again either i.m. or i.v. for up to 6 weeks, and outcomes were observed for up to 6 months. Of the 9 studies that assessed neurological outcomes, 8 reported significant effects in favor of GM1 (79–84, 86, 87) and one, i.e., the smallest trial with <20 patients, showed greater improvement with GM1 without reaching significance though (88). Of the 6 efficacy studies with brain imaging during follow-up (81–86), the 2 trials using PET showed a trend for an improvement of brain metabolism with GM1 (85, 86), whereas in none of the studies, CT revealed any morphological differences vs. placebo.

Except for one patient who stopped treatment due to an exfoliative dermatitis probably related to GM1, none of the 14 trials reported any major difference as compared to placebo regarding the frequency, nature, or severity of AEs.

Diabetes mellitus is a well-known risk factor for cerebral ischemia, and both acute hyperglycemia and chronic diabetes exacerbate ischemic brain damage (89). To determine if GM1 might be used as a neuroprotective agent in diabetes-associated cerebral ischemia/reperfusion injury, two *in vivo* studies in rats have investigated its effects in hyperglycemia-exacerbated ischemic brain damage. The first study indicated that GM1 attenuated diabetic-augmented ischemic neuronal injuries through the suppression of ERK1/2 phosphorylation (90). The second study showed that the attenuation of diabetes-associated cerebral ischemia/reperfusion injury by GM1 was related to the prevention of endoplasmic reticulum stress-induced apoptosis (91). These findings are in accordance with published results that have shown diabetes-enhanced ischemic brain damage is associated with activation of ERK1/2 (92) and augmentation of endoplasmic reticulum stress (93). Drugs that suppress ERK1/2 or elevate the endoplasmic reticulum stress have been reported to ameliorate brain damage in diabetic animals (94–96). These new experimental findings warrant further clinical investigation of GM1 particularly in the treatment of diabetes patients suffering ischemic stroke.

### Parkinson's Disease

PD is the second most common, progressive neurodegenerative disorder after AD, with 1–2 cases per 1,000 being affected at any time (97, 98). Clinically, the disease is characterized by bradykinesia, rigidity, resting tremor, gait disturbance, and postural instability as well as by cognitive, affective, and autonomic components. Pathological characteristics are α-synuclein-containing Lewy bodies and a loss of dopaminergic neurons in the substantia nigra and forebrain. In most cases, the cause of PD is unknown, however some genetic factors have been identified in 5–10% of patients (97) and several environmental factors have been shown to be associated with an increased risk of PD (99).

Although there are numerous clinical trials ongoing in PD, the unmet need for better symptomatic as well as disease-modifying therapies is still high. The most efficacious symptomatic treatment for PD is still the first drug approved, i.e., the combination of levodopa and a dopa decarboxylase inhibitor and no treatment has yet been shown to unequivocally slow disease progression. Development of therapies with neuroprotective/restorative effects is a rational approach for drug development in PD: the disease is slowly progressive and patients get worse over time; the dopaminergic reserve early in the disease (i.e., residual intact dopamine neurons) is a potential target for neuroprotection; damaged or dysfunctional, but still viable, dopamine neurons are targets for restoration.

Treatment of PD with GM1 may particularly make sense as the pathology of PD is multifactorial, including mechanisms such as Fas-mediated cell death, oxidative stress, mitochondrial dysfunction, cytoskeletal disruption, expression of inflammatory cytokines, ATP depletion, excitotoxicity, and loss of trophic support, among other possible factors. Accordingly, to achieve effective disease modification, multiple mechanisms may need to be targeted and GM1 is known to act through diverse mechanisms including inhibiting apoptosis, inflammation, excitotoxicity, and oxidative stress reactions, modulating calcium homeostasis, neurotrophic factor signaling, membrane integrity, cAMP levels, protein kinase activity, neuritogenesis, and axonogenesis (29, 48). Additionally, GM1 levels in neuronal plasma membranes may stabilize lipid raft signaling domains (100, 101) and intracellularly, may inhibit toxic synuclein aggregation (102).

GM1 was shown to rescue damaged dopaminergic neurons *in vitro* (103) and *in vivo*, to cause (a) increases in striatal dopamine levels and tyrosine hydroxylase-positive fiber density in the striatum and (b) reduced loss of pars compacta neurons in the substantia nigra in mice exposed to the Parkinson-producing neurotoxin 1-methyl-4-phenyl-1,2,3,6-tetrahydropyridine (MPTP) (104). These results were confirmed in MPTP-treated monkeys, which in addition to increased striatal dopamine levels and enhanced dopaminergic innervation of the striatum, also recovered from PD-like motor symptoms after treatment with GM1 (105). GM1 also increased the density of striatal dopamine transporter sites, suggestive of recovery or possibly sprouting of dopaminergic terminals. Based on these and other promising results in animal models of PD, GM1 went into clinical testing in PD patients.

**Figure 3:**
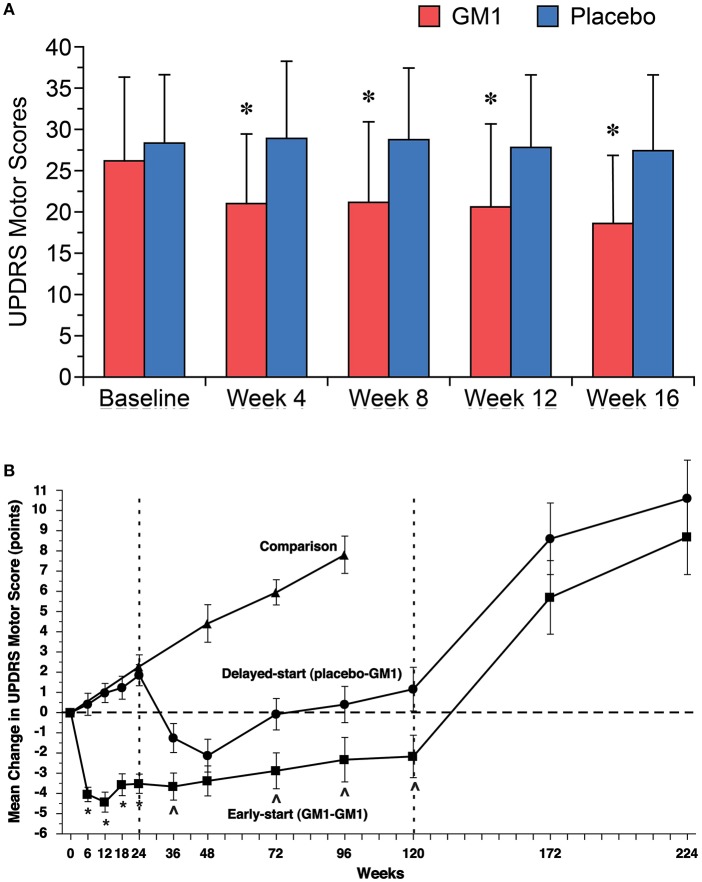
**(A)** Significant improvements in mean (±SD) Unified Parkinson's Disease Rating Scale (UPDRS) motor scores were noted in GM1-treated patients (red bars) beginning after 4 weeks of treatment and were maintained during the 16-week study. Mean UPDRS motor scores for the placebo group (blue bars) did not change significantly. Asterisks represent significant difference from baseline within the GM1 group. [Reprinted with permission from: Schneider et al. (106). https://n.neurology.org/content/neurology/50/6/1630.full]. **(B)** Changes in Unified Parkinson's Disease Rating Scale (UPDRS) Motor Subsection scores in a delayed start trial of GM1 in PD. The mean (±SE) change from baseline (observed scores) in Early-start and Delayed-start study subjects and in the standard-of-care Comparison group, assessed in the practically defined “off” condition. The dashed vertical line at week 24 indicates the end of study Phase I. The dashed vertical line at week 120 indicates the end of study Phase II. The horizontal dashed line indicates baseline level. An increase of score indicates symptom worsening; a decrease in score indicates symptom improvement. These data suggest a potential disease modifying effect of GM1 on PD. *p < 0.0001 Early-start vs. Delayed-start; *p* < 0.05 Early-start vs. Delayed-start. [Reprinted from Schneider et al. (107), with permission from Elsevier].

In a first, open-label study, effects of GM1 were tested in 10 PD patients who received 1,000 mg GM1 i.v. once after the last of three baseline functional assessments. Thereafter, patients self-administered GM1 at 200 mg/d s.c. for 18 weeks (7). There were no serious adverse events and none of the patients developed elevated anti-GM1 antibody titers. Most patients demonstrated improvements on at least some functional measures, beginning after 4–8 weeks of treatment. When functional improvements occurred, they lasted for the duration of the study. In a subsequent double-blind, randomized, placebo-controlled study, 45 patients with mild to moderate PD received either placebo or GM1, again with an i.v. loading dose of 1,000 mg followed by 200 mg/day s.c. for 16 weeks (Figure 3A) (106). The primary efficacy measure was the change in the Unified Parkinson's Disease Rating Scale (UPDRS) motor score, assessed at three independent baseline visits and then monthly while on treatment. At 16 weeks, there was a significant difference between treatment and placebo groups in UPDRS motor scores as well as in activities of daily living (ADL) scores. GM1-treated patients also had significantly greater improvements in performance of timed motor tests, including tests of arm, hand, and foot movements, and walking. GM1 was well tolerated and no serious adverse events were reported.

To evaluate long-term safety and efficacy of GM1, patients completing this 16-week trial were offered to enter an open-label extension study (108). Twenty-six patients received GM1 at 200 mg/day s.c. for up to 5 years. Safety was evaluated monthly and efficacy every 6 months. After 5 years, patients of the former placebo group improved in UPDRS motor, but not ADL scores. Patients treated with GM1 throughout both the double blind and the open-label extension study showed only a slight deterioration of UPDRS motor and ADL scores over 5 years, with both scores remaining significantly below those obtained at baseline prior to randomization into the original study. No relevant safety issues or changes in safety laboratory measurement were noted over the course of the study. Results suggested that long-term GM1 use in PD is safe and may have some disease modifying potential. That the patients treated with placebo during the double-blind study did not fully catch up to the patients who used GM1 during the double-blind study over the subsequent 5-year period is of particular interest, as this would have been expected for a purely symptomatic treatment.

These findings led to the conduct of another double-blind, randomized, placebo-controlled study using a delayed-start design to distinguish between potential symptomatic and disease modifying effects of GM1 in PD: 77 patients were randomized to receive either GM1 (early-start) for 120 weeks or placebo for the first 24 weeks and subsequently GM1 for 96 weeks (delayed-start); 17 additional patients received standard-of-care in order to follow the natural disease progression (Figure 3B) (107). At week 24, the early-start group demonstrated significant improvement in UPDRS motor scores vs. a significant worsening of scores in the delayed-start (placebo) group. The delayed start group showed improvements in UPDRS scores after starting GM1 after week 24. The early-start group showed a sustained benefit vs. the delayed-start group at week 72 and at week 120, and the trajectory of the two groups remained divergent at the end of the treatment period. Both groups had significant symptom worsening after 1 and 2 years of washout. The most prevalent AEs were injection site reactions and only 3 subjects reported serious adverse events (i.e., asthenia, worsening of PD symptoms, and anastomotic ulcer/stomach cancer). There were no consistent relevant changes in clinical chemistry. This study provided evidence that GM1 use for 24 weeks was superior to placebo in improving motor symptoms and that extended GM1 use (up to 120 weeks) resulted in a lower than expected rate of symptom progression. Thus, GM1 may not only have symptomatic effects on PD but may also have disease-modifying effects.

### Huntington's Disease

HD is an autosomal-dominant, progressive neurodegenerative disorder with the highest prevalence in the Caucasian population, with 7 per 100,000 being affected (109). The disease usually starts at around 40 years of age and progresses inexorably to death within 10–20 years. Patients with HD display characteristic choreic involuntary movements and impaired motor coordination, but also cognitive and psychiatric problems such as anxiety and depression, that often precede motor symptoms and are the most difficult to manage (110, 111).

The underlying cause of HD is the pathological expansion of a polyQ stretch at the N-terminus of huntingtin (HTT) (112), a ubiquitous scaffold protein with roles in vesicular traffic, autophagy, and transcriptional control of neural genes, among others (113). The HD mutation results in mutant HTT misfolding and aggregation, which in turn cause a plethora of cellular and network dysfunctions, leading first to changes in brain connectivity and generalized atrophy of the white matter, and then to neuronal death, mainly in regions that control movement, i.e., the striatum and the cerebral cortex (114).

Synthesis of gangliosides was shown to be decreased in cellular and animal models of HD (20, 21, 115) and in fibroblasts from HD patients (20). In HD cells, GM1 levels lower than normal correlated with increased susceptibility to apoptosis, suggesting a potential role in disease pathogenesis and/or progression (20). Administration of GM1 restored ganglioside levels and normal survival in HD cells *in vitro*, in part by increasing activation of the PI3K/AKT pathway and HTT phosphorylation (20). These initial observations prompted extensive pre-clinical *in vivo* studies to assess the therapeutic potential of ganglioside in HD.

**Figure 4:**
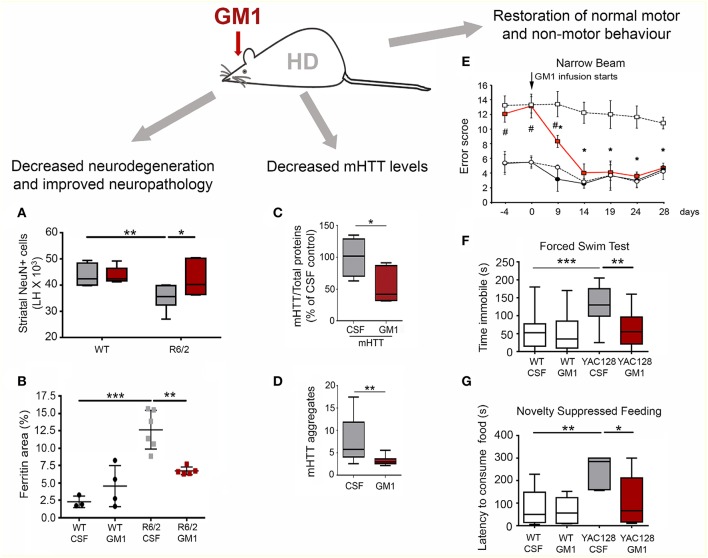
Disease-modifying effects of GM1 administration in HD mouse models. **(A)** Intraventricular infusion of GM1for 28 days resulted in a significant decrease in striatal neuron loss in the R6/2 mouse model of HD. LH, Left brain hemisphere. **(B)** Brain ferritin accumulation was attenuated in R6/2 mice treated with GM1. **(C)** Mutant HTT protein levels were decreased in the striatum of Q140 mice treated with GM1 for 42 days. **(D)** GM1 administration also attenuated accumulation of SDS-insoluble mHTT aggregates (as measured by a filter-trap assay). **(E)** GM1 administration resulted in restoration of normal motor function in YAC128 mice. Motor performance was scored as the mice walked along a narrow beam. **(F)** GM1 administration decreased depression-like behavior in YAC128 mice, as measured by the time mice spent immobile in a forced swim test. **(G)** In the novelty-suppressed feeding test, GM1 corrected anxiety-like behavior in YAC128 mice, as measured by the latency to consume sweetened condensed milk in a novel environment. Box-and-whisker plots show median, maximum and minimum values. *p < 0.05; **p < 0.001; ***p < 0.0001. **(A–D,F,G)** are reproduced with permission from Alpaugh et al. (120); **(E)** is reproduced with permission from Di Pardo et al. (119).

In line with rigorous NIH guidelines, GM1 was tested in three different - and for many aspects complementary—genetic models of HD, i.e., R6/2, Q140, and YAC128 mice (Figure 4) (116–118). Intraventricular infusion of GM1 for 28–42 days (depending on the animal model used) resulted in profound therapeutic and disease-modifying effects across all models (119, 120). Motor behavior was dramatically improved in R6/2 mice and restored to normal in YAC128 and Q140 mice, including gait abnormalities which are often resistant to treatments (119, 120). GM1 administration also corrected anxiety-like and depression-like behaviors, and improved cognitive functions in both YAC128 and Q140 mice (120). Phenotypic improvement upon treatment with GM1 correlated with profound disease-modifying effects. In R6/2 mice, which express a toxic N-terminal fragment of mutant HTT that causes widespread neuronal death and an accelerate disease phenotype (117), GM1 treatment slowed down neurodegeneration and decreased white matter atrophy and ferritin levels (120), which in HD patients correlate with iron accumulation and cortical and striatal atrophy (121, 122). In Q140 and YAC128 mice, GM1 administration restored normal expression and phosphorylation levels of dopamine- and cAMP-regulated neuronal phosphoprotein 32 (DARPP32) (119, 120), a key regulator of dopamine signaling and striatum output pathways (123, 124), suggesting that GM1 improved overall HD striatal function. Additional beneficial outcomes of GM1 administration were restoration of normal cortical levels of key neurotransmitters, including glutamate and GABA, modulation of dopamine and serotonin metabolism, and normal expression of glia markers (120).

Remarkably, GM1 treatment affected mutant HTT itself. Administration of GM1 increased HTT phosphorylation at Ser13 and Ser16 (119), a post-translational modification that was shown to decrease mutant HTT aggregation (125) and toxicity (125–127). Moreover, GM1 decreased levels of soluble and aggregated (SDS-insoluble) mutant HTT, without affecting HTT gene transcription and wild-type HTT levels (120). These important effects on mutant HTT, along with the more general neuroprotective activities described for GM1 (29, 30, 128), explain the widespread therapeutic effects of GM1 in HD models, which were comparable to (and in some cases exceeded) those observed in pre-clinical studies where antisense oligonucleotides were used to lower HTT levels (129).

In view of the wide therapeutic and disease-modifying effects of GM1 in HD models, clinical studies in this indication are certainly encouraged. To date, there is no cure or disease-modifying therapy for HD. Drug candidates in clinical development are either still in early phases or have failed to show benefits in HD patients (114, 130, 131). A Phase I/IIa clinical trial with antisense oligonucleotides (ASO) (ClinicalTrials.gov Identifier: NCT02519036) to lower HTT levels was recently completed and showed dose-dependent reduction of mutant HTT, prompting a Phase III trial to assess efficacy. While there is obvious enthusiasm toward the possibility to reduce, at least in part, mutant HTT levels in patients, important questions remain to be answered concerning long-term safety and the potential consequences of concomitantly lowering the levels of wild-type HTT, which has important functions in the nervous system (132). In light of these considerations, a combination therapy with ASOs and GM1 could be desirable and highly effective, by engaging additional pathways for mutant HTT clearance and neuroprotection, allowing for decreased ASO dosing and for extended therapeutic benefits.

### Alzheimer's Disease

AD is the most prevalent cause of dementia worldwide and remains a therapeutic challenge despite rapidly expanding research efforts (133). Given the important neuroprotective roles of brain gangliosides, GM1 was proposed as a therapeutic agent in AD (134). However, early clinical investigations of GM1 in AD were inconclusive (135): a double-blind, placebo-controlled trial of intramuscular GM1 did not find any significant cognitive amelioration (136); an uncontrolled study of intraventricular GM1 in five patients with AD showed marked improvements in several clinical outcomes (137). Nevertheless, these studies confirmed safety of GM1 administration even upon intraventricular administration.

AD is characterized by amyloid deposits, consisting mainly of aggregated variants of amyloid β (Aβ). The involvement of gangliosides in AD remains controversial. Early studies showed that GM1 binds to Aβ and seeds the conformational transition from random coil to an ordered structure rich in β-sheets (138), a pathological hallmark of the disease. Later experiments, however, suggested that this happens only at very high concentrations, while physiological levels of GM1 in an environment that mimics the composition of the neuronal plasma membrane inhibits the oligomerization of Aβ monomers driven by sphingomyelin (139). It remains to be determined whether high concentrations of GM1 with Aβ seeding can be achieved locally in specialized cellular compartments such as endosomes and synaptic terminals (140, 141). Studies in animal models support a non-detrimental or even a protective role of GM1 in AD.

In an AD mouse model with deletion of GD3 synthase (APP/PS-1/GD3S^−/−^), which lacked b-series gangliosides but had >50% increase in a-series gangliosides, including GM1 and GD1a, decreased accumulation of Aβ deposits and dramatically improved neuropathology and behavior were observed (142). Beneficial effects were also reported with administration of GM1 in the APP/PSEN-1 model of AD (143). At therapeutic concentrations, GM1 may help reducing overall Aβ load, sequestering excess Aβ in AD patients (144), activating autophagy to help with Aβ clearance (145), and by promoting Aβ elimination by microglia (146).

In conclusion, although the clinical experience of GM1 in AD is inconclusive, new insights into the neuroprotective potential of GM1 in AD might revitalize clinical research in this field.

## Perspectives

In summary, there is good evidence for faster recovery by GM1 in SCI, but the total extent of recovery about the same as without GM1. Therefore, there might be an adjunctive role only and it is not the highest medical need. Stroke is best investigated and numerous trials provided evidence of clinical efficacy of GM1; new clinical trials may only be needed for specific aspects. In PD, benefits are well documented, although studies need to be repeated in larger patient groups; other disease modifying therapies are under development. AD is highly prevalent and medical need for new treatment is high but the role of GM1 is still unclear and clinical data not conclusive. In HD, the medical need is high and preclinical results are convincing, but there are no clinical trials yet.

An important issue to be considered in the design of future clinical trials in neurodegenerative conditions is the route of drug administration and the extent to which GM1 would cross the blood-brain barrier. Although “central” neurotrophic effects of peripherally administered gangliosides have been shown in animal models of PD or stroke (105, 147, 148), and in PD patients by slowing down the loss of a marker for striatal dopaminergic terminals (149), it remains unclear whether GM1 can cross an intact blood-brain barrier (BBB) and reaches therapeutic concentrations in the human brain (150–153). In favor of the use of GM1 in various neurodegenerative/trauma-related indications is that the BBB is not entirely intact, so that more peripherally administered GM1 crosses a leaky BBB than predicted based on studies in models with an intact BBB. Perhaps, negative or inconclusive results in some of the past trials with GM1 might have been related to use of suboptimal dosages and/or lack of significant CNS penetration if the BBB was not compromised (154–156).

To facilitate the design of successful new clinical trials, distributions of the ganglioside to different brain regions upon administration through various routes should be carefully determined in relevant non-human primate disease models, as rodents often display different BBB permeability to various molecules (157, 158). Recent developments in strategies to overcome issues with drug delivery across the BBB might also help with GM1 CNS delivery in future studies.

## Author's Note

Lectures on the individual sections of this article were held on the International Workshop “Gangliosides in the twenty-first century: therapeutic prospects,” Washington, DC, November 10, 2017, convened by TRB Chemedica.

## Author Contributions

PM and HF: authored section Mechanisms of Action. FG: section Spinal Cord Injury. PL: section Stroke. JS: section Parkinson's Disease. SS: sections Huntington's Disease and Alzheimer's disease. All authors contributed to the final version of the review article.

### Conflict of Interest Statement

SS holds a non-provisional US patent for the use of GM1 in treating HD. PM advised TRB Chemedica for the organization of the workshop. The remaining authors declare that the research was conducted in the absence of any commercial or financial relationships that could be construed as a potential conflict of interest.
